# Effects of Three Traditional Chinese Fitness Exercises Combined with Antihypertensive Drugs on Patients with Essential Hypertension: A Systematic Review and Network Meta-Analysis of Randomized Controlled Trials

**DOI:** 10.1155/2021/2570472

**Published:** 2021-10-31

**Authors:** Lulu Dai, Yuerong Jiang, Peili Wang, Keji Chen

**Affiliations:** National Clinical Research Center for Chinese Medicine Cardiology, Xiyuan Hospital, China Academy of Chinese Medical Sciences, Beijing 100091, China

## Abstract

**Objective:**

To compare the efficacy of three different traditional Chinese exercises (Tai Chi, Baduanjin, and Wuqinxi) combined with antihypertensive drugs (AHD) on patients with essential hypertension (EH).

**Method:**

Eight electronic databases were searched to identify randomized controlled trials (RCTs) comparing the effects of traditional Chinese fitness exercises combined with AHD and AHD alone. The analysis mainly consists of network meta-analysis (NMA) and pairwise meta-analysis. The Cochrane assessment tool was adopted to assess the risk of bias of included literatures. This study used STATA/SE 15.1 (StataCorp, 2017), R software (version 4.0.1), and Cochrane's Review Manager software (version 5.4) to conduct data analysis and figures generation.

**Results:**

A total of 30 RCTs were included in this study, of which 16 evaluated Tai Chi plus AHD versus AHD, 11 evaluated Baduanjin plus AHD versus AHD, and 3 evaluated Wuqinxi plus AHD versus AHD. No RCT compared directly among the three traditional Chinese fitness exercises. Pairwise meta-analysis showed that Tai Chi plus AHD was significantly superior to AHD alone in reducing systolic blood pressure (SBP) and diastolic blood pressure (DBP). BDJ plus AHD was statistically superior to AHD alone in reducing SBP, DBP, and endothelin (ET) and increasing nitric oxide (NO). NMA results indicated that Tai Chi plus AHD (WMD −12.42 mmHg, 95% CI: −15.29 to −9.55) and Baduanjin plus AHD (WMD −7.03 mmHg, 95% CI: −9.80 to −4.26) were superior to AHD, and Tai Chi was more effective than other traditional exercises in lowering SBP, Tai Chi plus AHD (WMD −7.56 mmHg, 95% CI: −10.15 to −4.96) and Baduanjin plus AHD (WMD −4.51 mmHg, 95% CI: −7.38 to −1.65) were superior to AHD in reducing DBP, Baduanjin plus AHD (WMD 4.26 *μ*mol/L, 95%CI: 2.68 to 5.83) was statistically superior to AHD in increasing NO, and Tai Chi plus AHD (WMD −7.64 pg/ml, 95% CI: −10.46 to −4.83) and Baduanjin plus AHD (WMD −9.23 pg/ml, 95% CI: −10.85 to −7.61) were superior to AHD in lowering ET.

**Conclusion:**

*C*ompared with AHD alone, both Tai Chi plus AHD and Baduanjin plus AHD showed significant benefit in regulating SBP, DBP, and ET. Among the three traditional Chinese fitness exercises, Tai Chi may be the best as an adjunctive therapy for SBP reduction. These findings provided evidence for the therapeutic benefit of either Tai Chi or Baduanjin exercise as an adjunct therapy for patients with EH. Limited by the methodological quality and quantity of included studies, results need to be interpreted with caution, and it is necessary to carry out further high-quality RCTs on traditional Chinese fitness exercise-assisted treatment of EH in the future.

## 1. Introduction

The results of many cohort studies indicate that hypertension takes a leading role in the current global burden of cardiovascular disease and overall mortality [1, 2]. Data from the Global Burden of Disease project shows that nearly 9.4 million deaths every year are due to raised blood pressure [3]. Therefore, prevention of the occurrence and progress of hypertension disorders is a current global priority public health problem [4]. Pharmacotherapy of hypertension is an important tool for treatment of hypertension [5]. However, there are adverse drug reactions caused by AHD, especially in the elderly, drug-drug interactions may also increase the burden related to drugs, and adverse reactions related to AHD can lead to the suspension of drug treatment [6–8]. Consequently, pharmacotherapy of hypertension combined with non-pharmacological method is very important; non-pharmacological method is a complementary treatment of drug therapy and delay the need for pharmacotherapy [9]. Exercise is an important non-pharmacological method to prevent, treat, and control elevated blood pressure. Patients can better regulate blood pressure by following an appropriate fitness exercise prescription, which can promote overall health and improve quality of life; low- to moderate-intensity aerobic exercise is preferred for exercise therapy [10–14]. The 2017 Clinical Practice Guidelines for Hypertension recommends that adults diagnosed with hypertension or elevated blood pressure follow an organized exercise program to increase physical activity [15]. There have been some studies about exercise training practices on hypertension [10–14, 16], but comparatively little attention has been focused on exploring traditional Chinese fitness exercises as a complementary therapy for EH, such as Tai Chi, Baduanjin, and Wuqinxi. While there have been some meta-analyses on the effects of traditional Chinese fitness exercises as an adjuvant therapy for EH [17–26], relatively scarce studies provide data from direct comparisons between these traditional Chinese fitness exercises. Therefore, to address this gap, we intend to perform a network meta-analysis (NMA) to compare the effects of three different traditional Chinese fitness exercises combined with AHD with AHD alone on patients with EH. In contrast to traditional meta-analyses, which focus narrowly on a single-treatment comparison, a network meta-analysis is able to pool direct and indirect evidence, analyses all possible comparisons between all treatments for a disease, and assesses the relative merits of each treatment [27]. By means of NMA based on the frequentist framework, we could calculate surface under the cumulative ranking curve (SUCRA [28]) and the likelihood of being the best and the worst for each intervention to predict the curative effect ranking of each traditional Chinese fitness exercise, and provide direct information about the three types of traditional Chinese fitness exercises evidence.

## 2. Methods and Analysis

### 2.1. Search Strategy

Searches of the China Biology Medicine disc (CBM), China National Knowledge Infrastructure (CNKI), Wanfang Database (WANFANG), China Science and Technology Journal Database (CQVIP), Cochrane Central Register of Controlled Trials (CENTRAL), PubMed, EMBASE, and Web of Science were carried out for literatures published from journal inception to March 2021. There were no limitations in aspects of publication language or year in this search. The references in the relevant meta-analyses and systematic reviews and included literatures were reviewed carefully for relevant potential articles. PubMed search strategy is detailed in Table 1. For a complete search strategy of CENTRAL, PubMed, EMBASE, and Web of Science, be sure to read Supplementary Appendix 1.

### 2.2. Eligibility Criteria

#### 2.2.1. Inclusion Criteria

Inclusion criteria were (1) RCTs comparing Tai Chi or Baduanjin or Wuqinxi plus AHD with AHD alone; (2) RCTs enrolling adults (≥18 years) with EH (no restriction on gender, nation, or ethnic), according to the 2018 Chinese guidelines for the management of hypertension [29], the Seventh Report of the Joint National Committee on Prevention, Detection, Evaluation, and Treatment of High Blood Pressure (JNC 7) [30]; and (3) at least one of four interested outcome measures, which include SBP, DBP, NO, ET, and required to be documented in the literature.

#### 2.2.2. Exclusion Criteria

Exclusion criteria were (1) non-randomized controlled study; (2) participants with secondary hypertension or serious complications; (3) population or intervention not corresponding to our inclusion criteria; (4) no outcome measures of interest; (5) duplicated publications; (6) second publication of same trial; (7) conference abstract; (8) study protocol; (9) full text was not available; and (10) data cannot be extracted.

### 2.3. Study Selection

The retrieved literature records were managed by means of reference management software NoteExpress (version 3.2). We conducted the pilot selection of literature to make sure that the inter-rater reliability among assessors was high. Based on the inclusion and exclusion criteria, two independent researchers respectively conducted a detailed screening of titles and abstracts of reference records identified through database searching. All potential articles that meet the eligible criteria and controversial literatures were required for a full-text review. Arbitration will be carried out by the third researcher, who was responsible for resolving the confliction between the two researchers.

### 2.4. Data Extraction

Extraction of data of interest was separately conducted by two independent researchers after pilot extraction. The confliction between the two researchers would be resolved by the third researcher. The following data was what we need to extract: the first author of the research, publication year, diagnostic criteria for hypertension, level of blood pressure, sample, patient characteristics (age and sex), details of interventions, and outcome measures (SBP, DBP, NO, and ET).

### 2.5. Risk of Bias Appraisal and GRADE Assessment

Based on Cochrane Handbook 5.1.0 [31], two independent researchers separately reviewed the included literatures to assess the risk of bias. The methodological quality of included studies was classified as having a low, unclear, or high risk of bias. Any confliction of opinions during the appraisal process was resolved by the third researcher or panel discussion. Assessment items included the following 7 items: (1) random sequence generation, (2) allocation concealment, (3) blinding of participants and personnel, (4) blinding of outcome data assessment, (5) incomplete outcome data, (6) selective outcome data reporting, and (7) other bias. The Grading of Recommendations Assessment, Development and Evaluation (GRADE) approach was applied to appraise the quality of the evidence behind the ranking of interventions from NMA [32].

### 2.6. Statistical Analysis

#### 2.6.1. Pairwise Meta-Analyses

Cochrane's Review Manager software (version 5.4) was used to analyze continuous data. We use the mean difference (MD) and 95% CI for continuous variables. *I*^2^ values were used to evaluate the statistical heterogeneity between the included studies. When there is no or low heterogeneity between studies (*I*^2^ < 25%), we used fixed-effects model to conduct the meta-analysis. If there is substantial heterogeneity (25% < *I*^2^ < 95%) and clinical heterogeneity was considered acceptable, we used random-effects model to conduct the meta-analysis. When the statistical heterogeneity is particularly large (*I*^2^ > 95%) or clinical heterogeneity is particularly significant, quantitative data were not pooled.

#### 2.6.2. Network Meta-Analyses

This study used STATA/SE 15.1 (StataCorp, 2017) and R software (version 4.0.1) to conduct data analysis and figures generation. We used WMD and their associated 95% CIs to summarize results. We took into account the existence of heterogeneity among different RCTs; thus, the random-effects model was selected to combine effect sizes in this network meta-analysis. We used the node-splitting model to assess inconsistency between direct and indirect comparisons. The bias in publication and small-scale study effects were evaluated with comparison-adjusted funnel plots, which were generated using “netfunnel” command. The network geometry of three different traditional Chinese fitness exercises was shown and described with network evidence plots, which were generated using “networkplot” command. We calculated the SUCRA and likelihood of being the best and the worst for each intervention to predict the curative effect ranking of each traditional Chinese fitness exercise. The significance level for all data analyses of this network meta-analysis was predetermined at 0.05.

## 3. Results

### 3.1. Results of Study Selection

**Figure 1:**
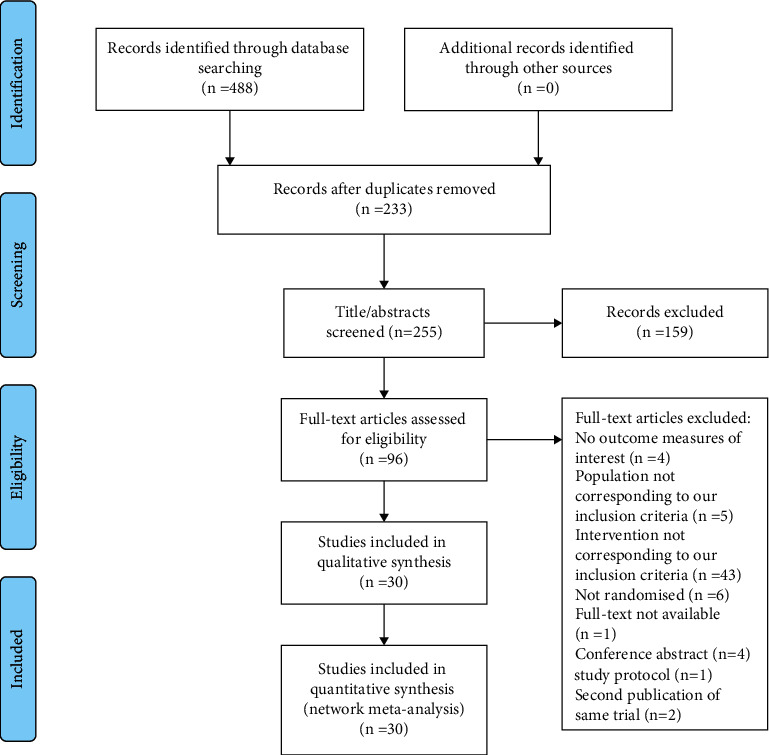
Flow diagram of literature identification and selection.

Initially, 488 records were identified through database searching. Then, duplicates were removed, and 255 records remained. 159 records were excluded after screening titles and abstracts. 96 studies were eligible for full-text screening. Finally, 30 studies met our inclusion criteria [26, 33–61]. The detailed selection process is illustrated in Figure 1.

### 3.2. Characteristics of Included Studies

Table 2 presents and describes the characteristics of included RCTs. This study involved 30 RCTs, 2160 participants with EH. All included RCTs were based on AHD-controlled two-arm studies. The years of publication of included RCTs were from 2006 to 2021. We included following three traditional Chinese fitness exercise types in our NMA: Tai Chi, Baduanjin, and Wuqinxi. 16 RCTs were Tai Chi interventions [26, 33, 34, 37, 40, 47–51, 54–58, 61], 11 RCTs were Baduanjin interventions [35, 36, 38, 39, 41, 42, 44, 46, 52, 59, 60], and the other 3 RCTs were Wuqinxi interventions [43, 45, 53]. The length of interventions ranged from 8 weeks to 5 years (260 weeks), most of which were 12 or 24 weeks.

### 3.3. Methodological Quality

**Figure 2:**
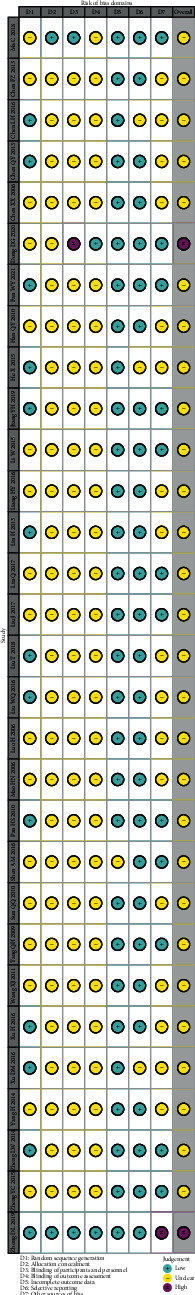
Traffic light plot of ROB.

**Figure 3:**
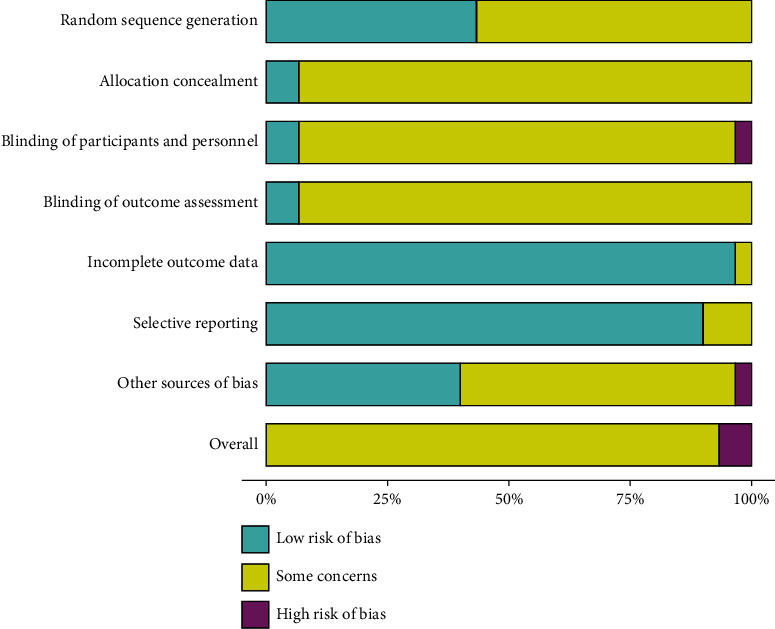
Summary plot of ROB.

Figures 2 and 3 present the risk of bias assessment of included RCTs. The overall methodological quality of included studies was low. In terms of random sequence generation, 13 [35, 36, 39, 41, 42, 45, 48, 49, 52, 57, 58, 60, 61] RCTs were judged as low risk and 17 [26, 33, 34, 37, 38, 40, 43, 44, 46, 47, 50, 51, 53–56, 59] were unclear risk. Twenty-eight [26, 34–60] RCTs were judged as unclear risk and 2 [33, 61] were judged as low risk in adequate allocation concealment. In terms of participants and personnel blinding, 27 [26, 34–37, 39–60] RCTs were judged as unclear risk and 2 [33, 61] showed low risk and 1 [38] was judged as high risk. In terms of outcome assessment blinding, 28 [26, 33–37, 39–60] RCTs were judged as unclear risk and 2 [38, 61] were low risk. Twenty-nine [26, 33–52, 54–61] RCTs were judged as low risk and only 1 [53] was unclear risk in incomplete outcome data. In terms of selective reporting, 3 [35, 41, 58] studies were judged as unclear risk, and 27 [26, 33, 34, 36–40, 42–57, 59–61] were low risks. In terms of other biases, 12 [26, 33, 38, 39, 42, 43, 46, 47, 52, 53, 55, 60] studies were judged as low risk and 17 [34–37, 40, 41, 44, 45, 48–51, 54, 56–59] were unclear risk and 1 [61] was high risk.

### 3.4. Results of Pairwise Meta-Analysis

Table 3 and Supplementary Appendix 2 show the results of pairwise meta-analysis. Tai Chi plus AHD was significantly superior to AHD alone in reducing SBP and DBP. BDJ plus AHD was statistically superior to AHD alone in reducing SBP, DBP, and ET and increasing NO. There were no statistically significant differences in other results.

### 3.5. Results of Network Meta-Analysis

#### 3.5.1. Assessment of Inconsistency

Lack of direct comparison resulted in no closed loop between different interventions, and all the included studies were 2-arm trials, so there was no need for inconsistency testing.

#### 3.5.2. Publication Bias

**Figure 4:**
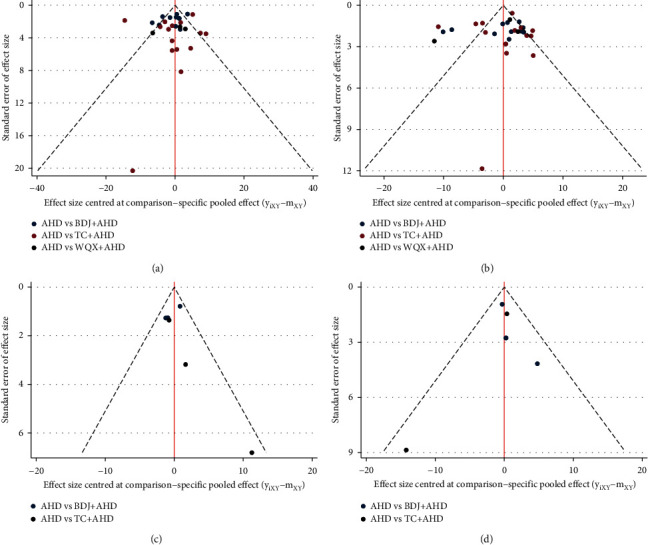
Comparison-adjusted funnel plots. The points of different colors represent a direct comparison of two interventions: (a) SBP; (b) DBP; (c) NO; and (d) ET.

The bias in publication and small-scale study effects are illustrated in Figure 4. Funnel plots for the SBP, NO, and ET network were roughly symmetrical, indicating that there was no apparent bias of publication due to small-scale study effects. The asymmetrical distribution of the funnel plots for the DBP network indicated that there was a possibility of bias of publication due to small-scale study effects.

#### 3.5.3. SBP Outcome

**Figure 5:**
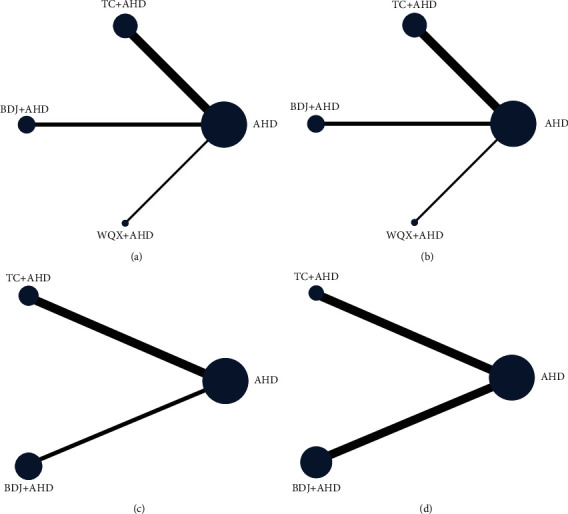
Network of direct comparisons formed by included studies. The node size and line thickness are proportionate to the number of studies: (A) SBP; (B) DBP; (C) NO; (D) ET.

**Figure 6:**
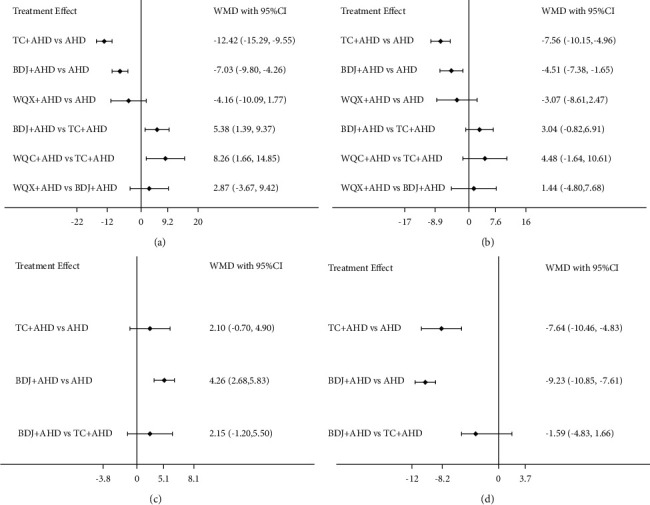
Effect size of different comparators presented in weighted mean difference (95% CI): (A) SBP; (B) DBP; (C) NO; (D) ET.

**Figure 7:**
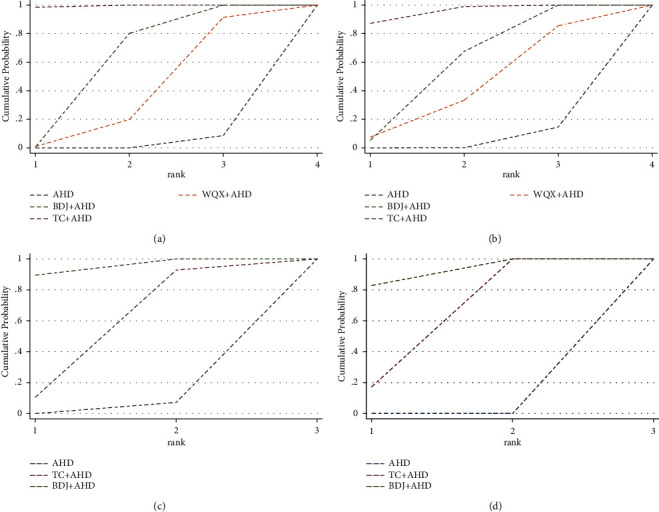
Cumulative ranking plots. Ranking of interventions are proportionate to the size of surface under the dotted line: (a) SBP; (b) DBP; (c) NO; (d) ET.

SBP was assessed in 29 trials (2100 participants). The network evidence plot for SBP is shown in Figure 5. In SBP reduction, Tai Chi plus AHD (WMD −12.42 mmHg, 95% CI: −15.29 to −9.55) and Baduanjin plus AHD (WMD −7.03 mmHg, 95% CI: −9.80 to −4.26) were superior to AHD alone, Baduanjin plus AHD (WMD 5.38 mmHg, 95% CI: 1.39 to 9.37) and Wuqinxi plus AHD (WMD 8.26 mmHg, 95% CI: 1.66 to 14.85) was statistically inferior to Tai Chi plus AHD; there was no statistically significant difference in other results (see Figure 6 and Table 4). According to SUCRA, the interventions to reduce SBP are ranked in probability (see Table 4 and Figure 7): TC + AHD (99.5%) > BDJ + AHD (60.2%) > WQX + AHD (37.4%) > AHD (2.9%). The quality of the evidence for the ranks of the treatment was low (see Table 5).

#### 3.5.4. DBP Outcome

DBP was assessed in 29 trials (2100 participants). The network evidence plot for DBP is shown in Figure 5. In DBP reduction, Tai Chi plus AHD (WMD −7.56 mmHg, 95% CI: −10.15 to −4.96) and Baduanjin plus AHD (WMD −4.51 mmHg, 95% CI: −7.38 to −1.65) were superior to AHD alone; there was no statistically significant difference in other results (see Figure 6 and Table 4). According to SUCRA, the interventions to reduce DBP are ranked in probability (see Table 4 and Figure 7): TC + AHD (95.4%) > BDJ + AHD (57.6%) > WQX + AHD (42.1%) > AHD (4.9%). The quality of the evidence for the ranks of the treatment was low (see Table 5).

#### 3.5.5. NO Outcome

NO was assessed in 6 RCTs (388 participants). The network evidence plot for NO is shown in Figure 5. In increasing NO, Baduanjin plus AHD (WMD 4.26 *μ*mol/L, 95% CI: 2.68 to 5.83) was superior to AHD alone; there was no statistically significant difference in other results (see Figure 6 and Table 4). According to SUCRA, the interventions to increase NO are ranked in probability (see Table 4 and Figure 7): BDJ + AHD (94.7%) > TC + AHD (51.7%) > AHD (3.6%). The quality of the evidence for the ranks of the treatment was low (see Table 5).

#### 3.5.6. ET Outcome

ET was assessed in 6 RCTs (390 participants). The network evidence plot for ET is shown in Figure 5. In ET reduction, Tai Chi plus AHD (WMD −7.64 pg/ml, 95% CI: −10.46 to −4.83) and Baduanjin plus AHD (WMD −9.23 pg/ml, 95% CI: −10.85 to −7.61) were statistically superior to AHD alone; there was no statistically significant difference in other results (see Figure 6 and Table 4). According to SUCRA, the interventions to decrease ET are ranked in probability (see Table 4 and Figure 7): BDJ + AHD (91.4%) > TC + AHD (58.6%) > AHD (0%). The quality of the evidence for the ranks of the treatment was low (see Table 5).

## 4. Discussion

### 4.1. Summary of Findings

The results indicated that combination therapies including Tai Chi plus AHD and Baduanjin plus AHD were superior to AHD alone, and Tai Chi was more effective than other traditional exercises at lowering SBP. Compared with AHD alone, Tai Chi plus AHD and Baduanjin Plus AHD produced a statistically significant DBP reduction. Compared with AHD alone, Baduanjin plus AHD produced a statistically significant NO increment, while that of Tai Chi plus AHD was not statistically significant. In terms of ET, both Baduanjin plus AHD and Tai Chi plus AHD produced a statistically significant reduction compared with AHD alone, while the difference between the two exercise types was not statistically significant.

Due to the lack of direct comparisons, there was no closed loop between different interventions, so node splitting method was not used to detect inconsistency; besides, the overall methodological quality of included studies was low, which increased the uncertainty of the results and required careful interpretation of the results.

### 4.2. Clinical Implications

According to previous research results, traditional Chinese fitness exercises (Tai Chi, Baduanjin, and Wuqinxi) as adjuvant therapy are relatively easy to learn and less intense, especially suitable for long-term training of elderly patients with EH, which can enhance the antihypertensive effect, reduce the dosage of antihypertensive drugs, and ease the economic burden [43, 57, 60, 62–65]. However, compared with antihypertensive drugs, the effects of traditional Chinese fitness exercises on EH remain understudied and evidence quality hierarchy of most of literatures in this field is at lower levels, meaning that it is necessary to carry out further high-quality randomized controlled trials on traditional Chinese fitness exercise-assisted treatment of EH in the future.

Based on the NMA results in this study, as adjunctive kinesiotherapy for EH, Tai Chi and Baduanjin have a statistically significant benefit in reducing the levels of SBP, DBP, and ET. Baduanjin has a statistically significant benefit in increasing the level of NO, while Wuqinxi may be the least effective. These findings of this NMA may be useful for clinicians and health professionals to select appropriate traditional Chinese fitness exercise as an adjuntive kinesiotherapy or early intervention prescription for patients with EH.

### 4.3. Strength and Limitations

There has been an increasing number of studies on traditional Chinese fitness exercises as adjuvant therapies for EH in recent years. However, our previous study rarely directly compared the effectiveness of different traditional Chinese fitness exercises. Traditional meta-analysis cannot compare multiple interventions, but NMA can address this issue. NMA attempts to integrate decision-making evidences through evaluating relative effectiveness of two or more alternative interventions in the same situation [66]. Consequently, in this study, we not only compared the effectiveness of AHD alone with traditional Chinese fitness exercises combined with AHD, but also calculated cumulative rankings for identifying superiority among the three traditional Chinese fitness exercises using Stata program based on the frequentist framework. This work could provide guidance and information for clinicians to choose traditional Chinese exercises as an adjunct therapy for patients with EH.

However, several limitations in this NMA should be noted. First, among the included literatures, there were only three literatures about Wuqinxi as an adjuvant therapy for EH, which was less than Tai Chi and Baduanjin. The estimates for the efficacy of Wuqinxi as adjuvant therapy for EH were open to considerable uncertainty as the number of studies was small, which leads to the current evidence and potential findings that still require careful interpretation. Second, since no RCT compared directly among the three traditional Chinese fitness exercises currently, we could only use indirect evidence to compare the efficacy of three traditional Chinese exercises. If the distribution of effect modifiers between different direct comparisons is unbalanced, the associated indirect comparisons will be biased [67]. Third, due to insufficient sample size of some exercise trials, confounding factors cannot be adequately controlled, leading to relatively unreliable estimates of therapeutic effects. Fourth, the quality of evidence in this NMA was low according to GRADE criteria. Therefore, if high-quality evidence is obtained, the effect ranking order of interventions and pooled effect sizes may change. Fifth, although the current review is not registered, which may lead to potential bias, we still strictly follow the steps of the system review.

## 5. Conclusions

Based on the current results, we can reach the following conclusion. Compared with AHD alone, both Tai Chi plus AHD and Baduanjin plus AHD show significant benefit in regulating SBP, DBP, and ET. Among three traditional Chinese fitness exercises, Tai Chi may be the best as an adjunctive therapy for SBP reduction. These findings provide evidence for the therapeutic benefit of either Tai Chi or Baduanjin exercise as an adjunct therapy for patients with EH. The overall methodological quality of included studies was low, so current results need to be interpreted with caution, and it is necessary to carry out further high-quality RCTs on traditional Chinese fitness exercise-assisted treatment of EH in the future.

## Figures and Tables

**Table 1:** Search strategy in PubMed.

Search	Query
#1	Search: (“Tai Ji”[Mesh]) OR (Tai-ji[Title/Abstract]) OR (Tai Chi[Title/Abstract]) OR (Chi, Tai[Title/Abstract]) OR (Tai Ji Quan[Title/Abstract]) OR (Ji Quan, Tai[Title/Abstract]) OR (Quan, Tai Ji[Title/Abstract]) OR (Taiji[Title/Abstract]) OR (Taijiquan[Title/Abstract]) OR (T'ai Chi[Title/Abstract]) OR (Tai Chi Chuan[Title/Abstract])
#2	Search: (Wuqinxi[Title/Abstract]) OR (Baduanjin[Title/Abstract])
#3	Search: (“Hypertension”[Mesh]) OR (Blood Pressure, High[Title/Abstract]) OR (Blood Pressures, High[Title/Abstract]) OR (High Blood Pressure[Title/Abstract]) OR (High Blood Pressures[Title/Abstract])
#4	Search: randomized controlled trial[Publication Type] OR randomized[Title/Abstract] OR placebo[Title/Abstract]
#5	Search: #1 OR #2
#6	Search: #3 AND #4 AND #5

**Table 2:** Characteristic of included studies.

Author	Diagnosis criteria	level of blood pressure	Sample N/M/F	Mean age (years)	Interventions	Exercise prescription	Length of intervention (weeks)	Outcome measures
Ma, 2018	NA	NA	55/−/−	69 ± 9.37	TC + AHD	3∼5 times/week, at least 60 mins/session	24	SBP; DBP
			58/−/−		AHD			
Chen, 2013	NA	NA	50/−/−	30∼82	TC + AHD	6 times/week, 30 mins/session	12	SBP; DBP
			18/−/−		AHD			
Chen, 2016	CGMH-2010	NA	28/15/13	69.98 ± 3.11	BDJ + AHD	5 times/week, 60 mins/session	12	SBP; DBP
			28/14/14	70.29 ± 1.77	AHD			
Chen, 2013	CGMH-2005	EH1	27/13/14	69.23 ± 3.72	BDJ + AHD	5times/week, 30 mins/session	12	SBP; DBP; NO; ET
			28/16/12	70.06 ± 3.51	AHD			
Chen, 2006	CGMH-2005	EH2, 3	2020/9/11	64.3	TC + AHD	1 time/day, 40 mins/session	10	SBP; DBP; NO
			20/13/7	60.7	AHD			
Dong, 2020	CGMH-2005	NA	21/−/−	30∼65	BDJ + AHD	5 times/week, 60 mins/session	16	SBP; DBP; ET
			21/−/−		AHD			
Fan, 2021	CGMH-2018	EH1, 2	38/21/17	71.87 ± 0.76	BDJ + AHD	5 days/week, 30 mins/session	12	SBP; DBP
			38/19/19	71.95 ± 0.97	AHD		12	
Han, 2010	1999 WHO-ISH	EH1, 2	30/−/−	62.12 ± 10.51	TC + AHD	1∼2 times/day, 45∼60 mins/session	260	SBP; DBP
			28/−/−		AHD			
He, 2015	CGMH-2005	EH1	42/22/20	68.51 ± 2.97	BDJ + AHD	5 times/week, 30 mins/session	12	SBP; DBP
			42/23/19	69.24 ± 2.45	AHD			
Jiang, 2019	CGMH-2010	EH1, 2	50/25/25	64.67 ± 3.15	BDJ + AHD	At least 5 days/week, 2 times/day, 30 mins/session	12	SBP; DBP
			50/26/24	65.23 ± 3.23	AHD		12	
Li, 2015	CGMH-2004	EH1, 2	30	NA	WQX + AHD	5∼6 times/week, 30 mins/session	24	SBP; DBP
			30		AHD			
Liang, 2016	CGMH-2010	NA	30/17/13	68.1 ± 10.1	BDJ + AHD	2 times/day, 20 mins/session	12	SBP; DBP
			30/16/14	70.5 ± 10.2	AHD			
Lin, 2013	WHO	EH1, 2	68/31/37	NA	WQX + AHD	6 times/week, 30 mins/session	24	SBP; DBP
			59/27/32		AHD			
Lin, 2017	CGMH-2010	EH1	58/−/−	58 ± 7.48	BDJ + AHD	5 days/week, 1 time/day, 30∼40 mins/session	24	SBP; DBP; NO; ET
			58/−/−		AHD			
Liu, 2017	NA	EH1, 2	80/47/33	43 ± 6.57	TC + AHD	2 times/day, more than 40 mins/session	24	SBP; DBP
			77/48/29	42.6 ± 5.67	AHD			
Liu, 2018	CGMH-2010	EH1, 2	35/18/17	62.4 ± 2.4	TC + AHD	1 time/day, 40∼60 mins/session	24	SBP; DBP
			35/19/16	63.1 ± 2.1	AHD			
Liu, 2016	CGMH-2015	EH1, 2	30/16/14	56.33 ± 7.16	TC + AHD	5 times/week, 40 mins/session	12	NO; ET
			30/19/11	56.80 ± 6.78	AHD			
Luo, 2006	1999 WHO-ISH	NA	44/24/20	44.74 ± 12.10	TC + AHD	1 time/day, 45 mins/session	24	SBP; DBP
			40/21/19	44.86 ± 13.05	AHD		24	
Mao, 2006	1999 WHO-ISH	NA	51/13/38	45∼70	TC + AHD	6 times/week, 60 mins/session	8	SBP; DBP; NO; ET
			2011/2/9	52∼72	AHD			
Pan, 2010	1999 WHO-ISH	EH1	24/14/10	62.1 ± 5.8	BDJ + AHD	5 days/week, 2 times/day, 45 mins/session	24	SBP; DBP
			24/13/11	61.4 ± 7.1	AHD			
Shen, 2016	CGMH-2013	EH1, 2	36/−/−	NA	WQX + AHD	6 times/week, 60 mins/session	24	SBP; DBP
			33/−/−		AHD			
Sun, 2010	CGMH-2005	EH1, 2	1932/7/25	57.19 ± 8.09	TC + AHD	6 times/week, 90 mins/session	12	SBP; DBP
			1932/10/22	57.25 ± 5.63	AHD			
Tang, 2009	1999 WHO-ISH	EH1, 2	2016/10/6	63.65 ± 8.71	TC + AHD	3∼5 times/week, 30∼60 mins/session	24	SBP; DBP
			2016/9/7	62.79 ± 7.43	AHD			
Wang, 2011	CGMH-2005	EH1, 2	30/−/−	NA	TC + AHD	5 times/week, 60 mins/session	16	SBP; DBP
			30/−/−		AHD			
Xu, 2016	1999 WHO-ISH	EH1, 2	30/17/13	38.07 ± 8.09	TC + AHD	2 times/day, 10 mins/session	8	SBP; DBP
			30/18/12	37.63 ± 9.09	AHD			
Xu, 2016	1999 WHO-ISH	EH1	50/25/25	69.38 ± 7.41	TC + AHD	NA	8	SBP; DBP
			50/26/24	69.54 ± 7.37	AHD			
Yang, 2014	CGMH-2010	140 mmHg ≤ SBP <170 mmHg, or 90 mmHg ≤ DBP	33/13/20	60.07 ± 5.84	BDJ + AHD	5 times/week, 40 mins/session	24	SBP; DBP
		<110 mmHg	34/16/18	60.60 ± 7.37	AHD			
Zheng, 2014	CGMH-2005	EH1	27/13/14	69.23 ± 3.72	BDJ + AHD	5 days/week, 1 time/day 30 mins/session	12	SBP; DBP; NO; ET
			28/16/12	70.06 ± 3.51	AHD			
Zheng, 2015	CGMH-2010	NA	49/20/29	54.71 ± 5.43	TC + AHD	4∼8 times/week, 40∼60 mins/session	12	SBP; DBP
			49/20/29	55.77 ± 6.24	AHD			
Zhong, 2019	CGMH-2010	EH1, 2	2010/2/8	66.8 ± 3.26	TC + AHD	3 times/week, 60 mins/session	12	SBP; DBP
			2009/4/5	66.22 ± 3.27	AHD			

SBP: systolic blood pressure; DBP: diastolic blood pressure; NO: nitric oxide; ET: endothelin; AHD: antihypertensive drugs; TC + AHD: Tai Chi plus antihypertensive drugs; BDJ + AHD: Baduanjin plus antihypertensive drugs; WQX + AHD: Wuqinxi plus antihypertensive drugs; EH: essential hypertension; CGMH: Chinese Guidelines for the Management of Hypertension; and NA: not available.

**Table 3:** Results of the pairwise meta-analysis.

Comparison	*n*	SBP	*n*	DBP	*n*	NO	*n*	ET
		MD (95% CI)		MD (95% CI)		MD (95% CI)		MD (95% CI)
TC + AHD vs AHD	15	–**12.25 (**–**16.22 to –8.28)**	15	**–7.58 (–9.98 to –5.19)**	3	3.05 (–1.32 to 7.43)	2	–11.94 (–25.29 to 1.41)
BDJ + AHD vs AHD	11	**–6.92 (–8.70 to –5.14)**	11	**–4.50 (–7.08 to –1.92)**	3	**4.42 (3.26 to 5.58)**	4	**–9.23 (–10.85 to –7.61)**
WQX + AHD vs AHD	3	–4.10 (–9.39 to 1.19)	3	–3.23 (–9.98 to 3.52)	—		—	

SBP: systolic blood pressure; DBP: diastolic blood pressure; NO: nitric oxide; ET: endothelin; AHD: antihypertensive drugs; TC + AHD: Tai Chi plus antihypertensive drugs; BDJ + AHD: Baduanjin plus antihypertensive drugs; WQX + AHD: Wuqinxi plus antihypertensive drugs; MD: mean difference; and CI: credible intervals. The bold values mean that the results are statistically significant.

**Table 4:** Results of network meta-analysis of SBP, DBP, NO, and ET.

Treatment type	No adjustment	Likelihood (%) of being			SUCRA (%)
	Pooled WMD (95% Cl)	*P* value	Best	Worst	
SBP					
AHD	Reference	—	0	91.5	2.9
TC + AHD	−12.42(–15.29 to –9.55)	**<0.001**	98.5	0	99.5
BDJ + AHD	–7.03(–9.80 to –4.26)	**<0.001**	0.5	0	60.2
WQX + AHD	–4.16(–10.09 to 1.77)	0.169	1	8.6	37.4
DBP					
AHD	Reference	—	0	85.4	4.9
TC + AHD	–7.56 (–10.15 to –4.96)	**<0.001**	87.2	0	95.4
BDJ + AHD	–4.51 (–7.38 to –1.65)	**0.002**	5.3	0.1	57.6
WQX + AHD	–3.07 (–8.61 to 2.47)	0.277	7.5	14.5	42.1
NO					
AHD	Reference	—	0	92.8	3.6
TC + AHD	2.10 (–0.70 to 4.90)	0.141	10.5	7.2	51.7
BDJ + AHD	4.26 (2.68 to 5.83)	**<0.001**	89.5	0	94.7
ET					
AHD	Reference	—	0	100	0
TC + AHD	–7.64 (–10.46 to –4.83)	**<0.001**	17.2	0	58.6
BDJ + AHD	–9.23 (–10.85 to –7.61)	**<0.001**	82.8	0	91.4

SBP: systolic blood pressure; DBP: diastolic blood pressure; NO: nitric oxide; ET: endothelin; AHD: antihypertensive drugs; TC + AHD: Tai Chi plus antihypertensive drugs; BDJ + AHD: Baduanjin plus antihypertensive drugs; WQX + AHD: Wuqinxi plus antihypertensive drugs; WMD: weighted mean difference; CI: credible intervals; and SUCRA: surface under the cumulative ranking curve. The bold values mean that the results are statistically significant.

**Table 5:** Summary of confidence in ranking of interventions for outcomes (GRADE approach) in studies.

Outcome	Confidence	Downgrading due to
SBP	Low	Study limitations; indirectness
DBP	Low	Study limitations; indirectness
NO	Low	Study limitations; indirectness
ET	Low	Study limitations; indirectness

SBP: systolic blood pressure; DBP: diastolic blood pressure; NO: nitric oxide; and ET: endothelin.

## Data Availability

The datasets used during the current study are available from the corresponding author upon reasonable request.

## Supplementary Materials

Supplementary Materials
*Supplementary Appendix 1*. Search strategies of CENTRAL, PubMed, EMBASE, and Web of Science. *Supplementary Appendix 2*. Forest plots of pairwise meta-analysis of effect of traditional Chinese exercise on essential hypertension.
