# Supplementary material for: Effects of Three Traditional Chinese Fitness Exercises Combined with Antihypertensive Drugs on Patients with Essential Hypertension: A Systematic Review and Network Meta-Analysis of Randomized Controlled Trials
Source: Evid Based Complement Alternat Med. 2021 Oct 31;2021:2570472. doi: 10.1155/2021/2570472 (PMC8572593; doi:10.1155/2021/2570472)
Supplement: Supplementary Materials — Supplementary Appendix 1. Search strategies of CENTRAL, PubMed, EMBASE, and Web of Science. Supplementary Appendix 2. Forest plots of pairwise meta-analysis of effect of traditional Chinese exercise on essential hypertension. [file 2570472.f1.zip › 2570472.f1/Supplementary Appendix 1.docx]

Supplementary Appendix 1.

**Search strategy of CENTRAL, PubMed, EMBASE and Web of Science.**

| **Table 1: Search strategy in PubMed** | |
| --- | --- |
| Search | Query |
| #1 | Search: ("Tai Ji"[Mesh]) OR (Tai-ji[Title/Abstract]) OR (Tai Chi[Title/Abstract]) OR (Chi, Tai[Title/Abstract]) OR (Tai Ji Quan[Title/Abstract]) OR (Ji Quan, Tai[Title/Abstract]) OR (Quan, Tai Ji[Title/Abstract]) OR (Taiji[Title/Abstract]) OR (Taijiquan[Title/Abstract]) OR (T'ai Chi[Title/Abstract]) OR (Tai Chi Chuan[Title/Abstract]) |
| #2 | Search: (Wuqinxi[Title/Abstract]) OR (Baduanjin[Title/Abstract]) |
| #3 | Search: ("Hypertension"[Mesh]) OR (Blood Pressure, High[Title/Abstract]) OR (Blood Pressures, High[Title/Abstract]) OR (High Blood Pressure[Title/Abstract]) OR (High Blood Pressures[Title/Abstract]) |
| #4 | Search: randomized controlled trial[Publication Type] OR randomized[Title/Abstract] OR placebo[Title/Abstract] |
| #5 | Search: #1 OR #2 |
| #6 | Search: #3 AND #4 AND #5 |

| **Table 2: Search strategy in Embase** | |
| --- | --- |
| Search | Query |
| #1 | 'hypertension'/exp |
| #2 | 'blood pressure, high':ab,ti OR 'blood pressures, high':ab,ti OR 'high blood pressure':ab,ti OR 'high blood pressures':ab,ti |
| #3 | #1 OR #2 |
| #4 | 'tai chi'/exp |
| #5 | 'tai-ji':ab,ti OR 'tai chi':ab,ti OR 'chi, tai':ab,ti OR 'tai ji quan':ab,ti OR 'ji quan, tai':ab,ti OR 'quan, tai ji':ab,ti OR 'taiji':ab,ti OR 'taijiquan':ab,ti OR 'tai chi chuan':ab,ti |
| #6 | 'wuqinxi':ab,ti OR 'baduanjin':ab,ti |
| #7 | #4 OR #5 OR #6 |
| #8 | randomized controlled trial':ab,ti OR 'randomized':ab,ti OR 'placebo':ab,ti |
| #9 | #3 AND #7 AND #8 |

| **Table 3: Search strategy in Web of science** | |
| --- | --- |
| Search | Query |
| # 1 | TS= (Tai Chi OR Tai-ji OR Tai Chi OR Chi, Tai OR Tai Ji Quan OR Ji Quan, Tai OR Quan, Tai Ji OR Taiji OR Taijiquan OR T'ai Chi OR Tai Chi Chuan OR Wuqinxi OR Baduanjin) |
| # 2 | TS= (Hypertension OR Blood Pressure, High OR Blood Pressures, High OR High Blood Pressure OR High Blood Pressures) |
| # 3 | TS= (randomized controlled trial OR randomized OR placebo) |
| # 4 | #1 AND #2 AND #3 |

| **Table 4: Search strategy in Cochrane** | |
| --- | --- |
| Search | Query |
| #1 | MeSH descriptor: [Hypertension] explode all trees |
| #2 | (Blood Pressure, High):ab,ti,kw OR (Blood Pressures, High):ab,ti,kw OR (High Blood Pressure):ab,ti,kw OR (High Blood Pressures):ab,ti,kw |
| #3 | MeSH descriptor: [Tai Ji] explode all trees |
| #4 | (Tai-ji):ab,ti,kw OR (Tai Chi):ab,ti,kw OR (Chi, Tai):ab,ti,kw OR (Tai Ji Quan):ab,ti,kw OR (Ji Quan, Tai):ab,ti,kw OR (Quan, Tai Ji):ab,ti,kw OR (Taiji):ab,ti,kw OR (Taijiquan):ab,ti,kw OR (T'ai Chi):ab,ti,kw OR (Tai Chi Chuan):ab,ti,kw OR (Wuqinxi):ab,ti,kw OR (Baduanjin):ab,ti,kw |
| #5 | #1 or #2 |
| #6 | #3 or #4 |
| #7 | #5 and #6 |
