# Supplementary material for: Effects of Three Traditional Chinese Fitness Exercises Combined with Antihypertensive Drugs on Patients with Essential Hypertension: A Systematic Review and Network Meta-Analysis of Randomized Controlled Trials
Source: Evid Based Complement Alternat Med. 2021 Oct 31;2021:2570472. doi: 10.1155/2021/2570472 (PMC8572593; doi:10.1155/2021/2570472)

Supplementary Appendix 2.

Forest plots of pairwise meta-analysis of effect of traditional Chinese exercise for essential hypertension.

1 **TC+AHD vs AHD**

- 1. **SBP**


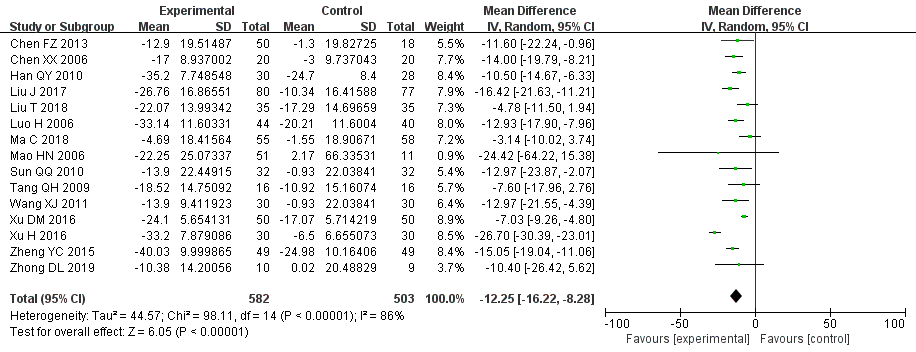


- 1. **DBP**


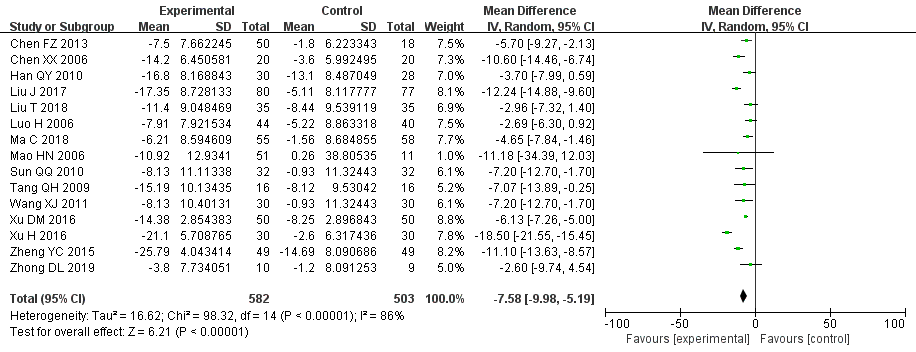


- 1. **NO**


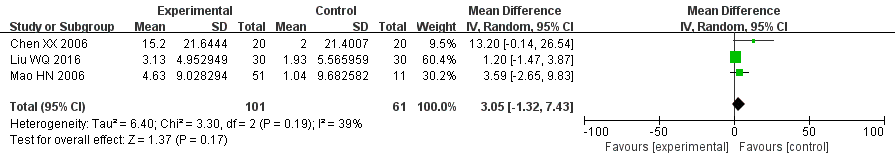


- 1. **ET**


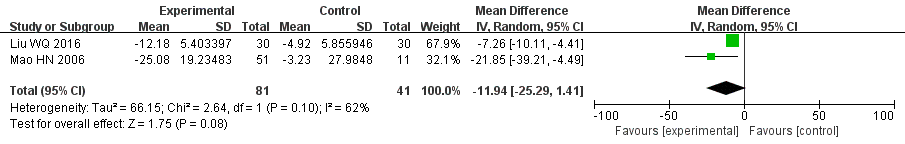


**2 BDJ+AHD vs AHD**

**2.1 SBP**


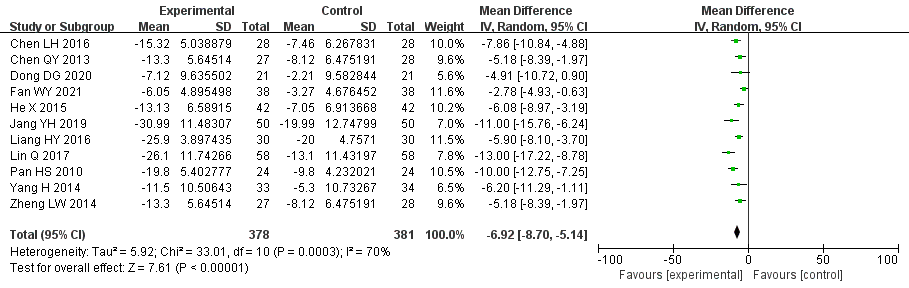


**2.2 DBP**


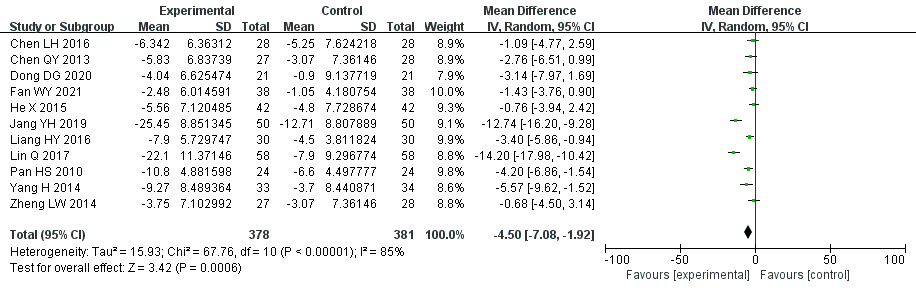


**2.3 NO**


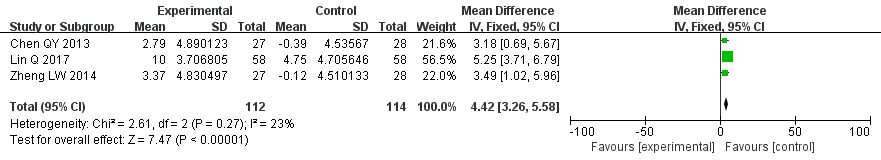


**2.4 ET**


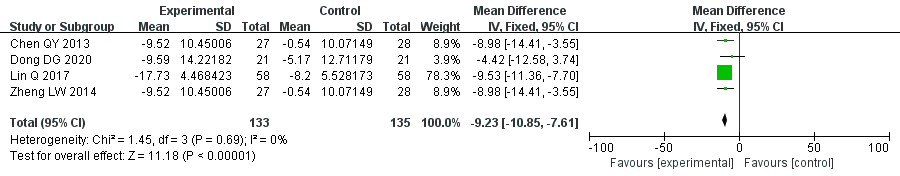


**3 WQX+AHD vs AHD**

**3.1 SBP**


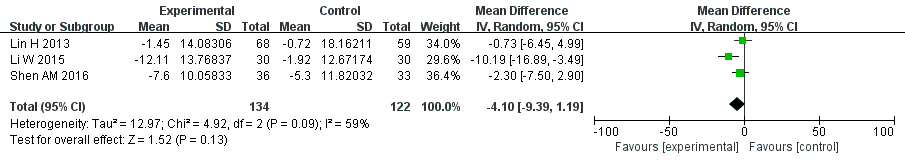


**3.2 DBP**


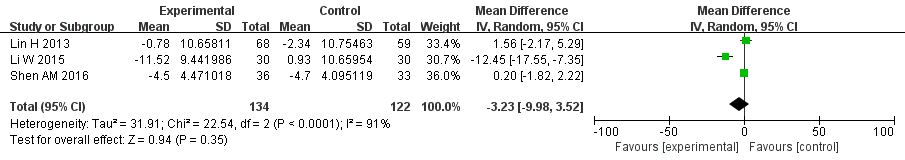

Supplement: Supplementary Materials — Supplementary Appendix 1. Search strategies of CENTRAL, PubMed, EMBASE, and Web of Science. Supplementary Appendix 2. Forest plots of pairwise meta-analysis of effect of traditional Chinese exercise on essential hypertension. [file 2570472.f1.zip › 2570472.f1/Supplementary Appendix 2.docx]
